# Supplementary material for: The Genomic Evolution and the Transmission Dynamics of H6N2 Avian Influenza A Viruses in Southern China
Source: Viruses. 2022 May 26;14(6):1154. doi: 10.3390/v14061154 (PMC9229805; doi:10.3390/v14061154)
Supplement: Supplementary file 1 [file viruses-14-01154-s001.zip › Supplementary Figure S1.pdf]

HA

Figure S1. Phylogenic trees of H6 subtype influenza viruses using the genomic sequences of HA, NA, PB2, PB1, PA, NP, M, and NS genes. The scale bar represents the number of nucleotide substitutions per site (subs/site).

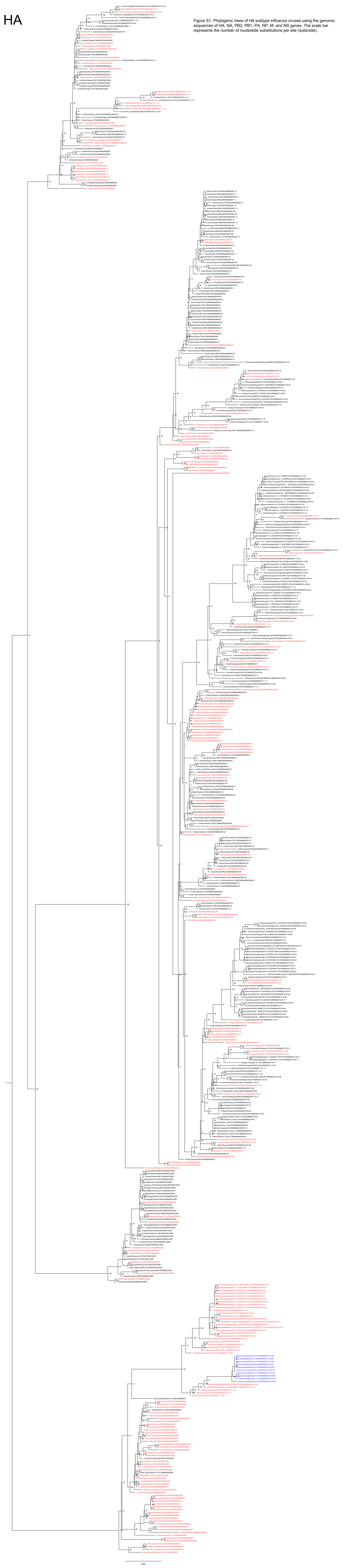

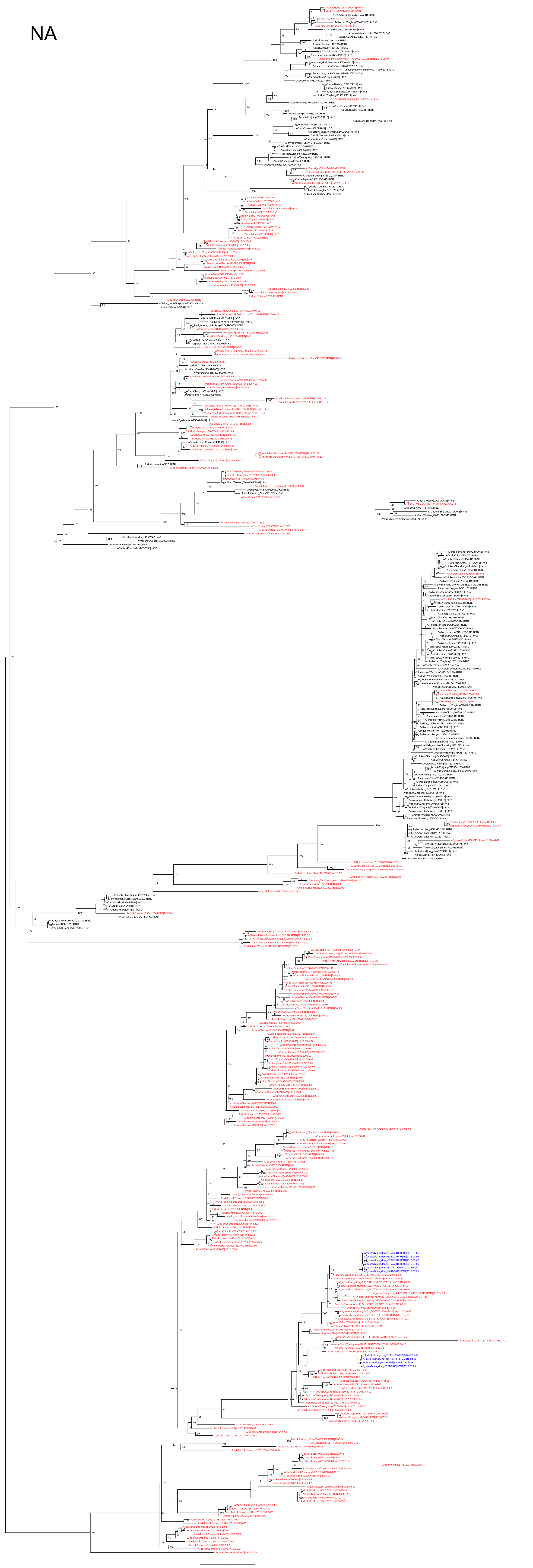
NA
0.02

PB2

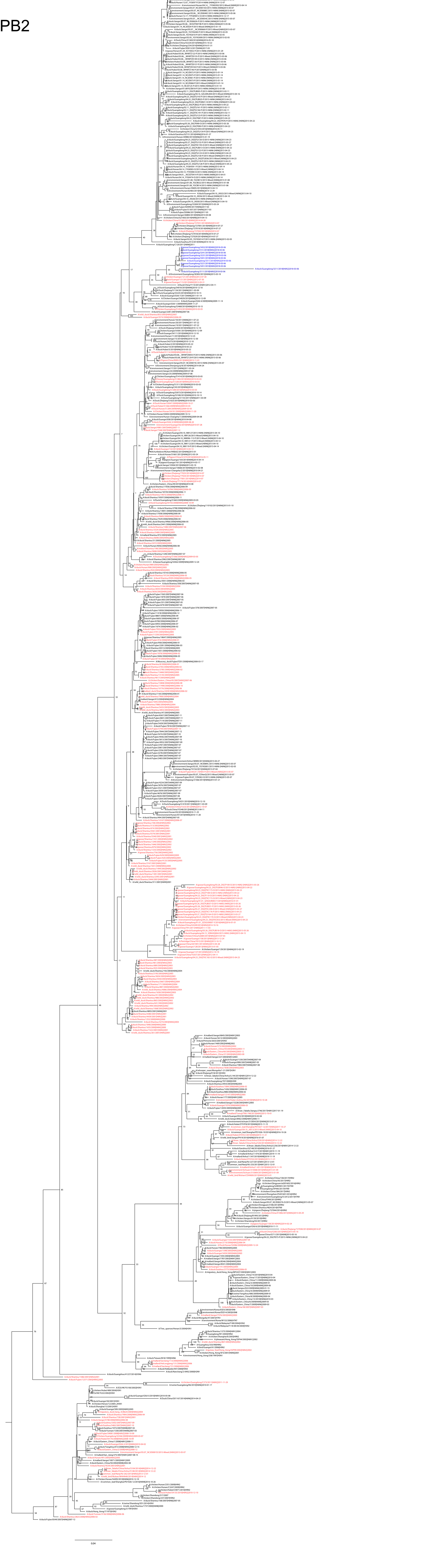

PB1

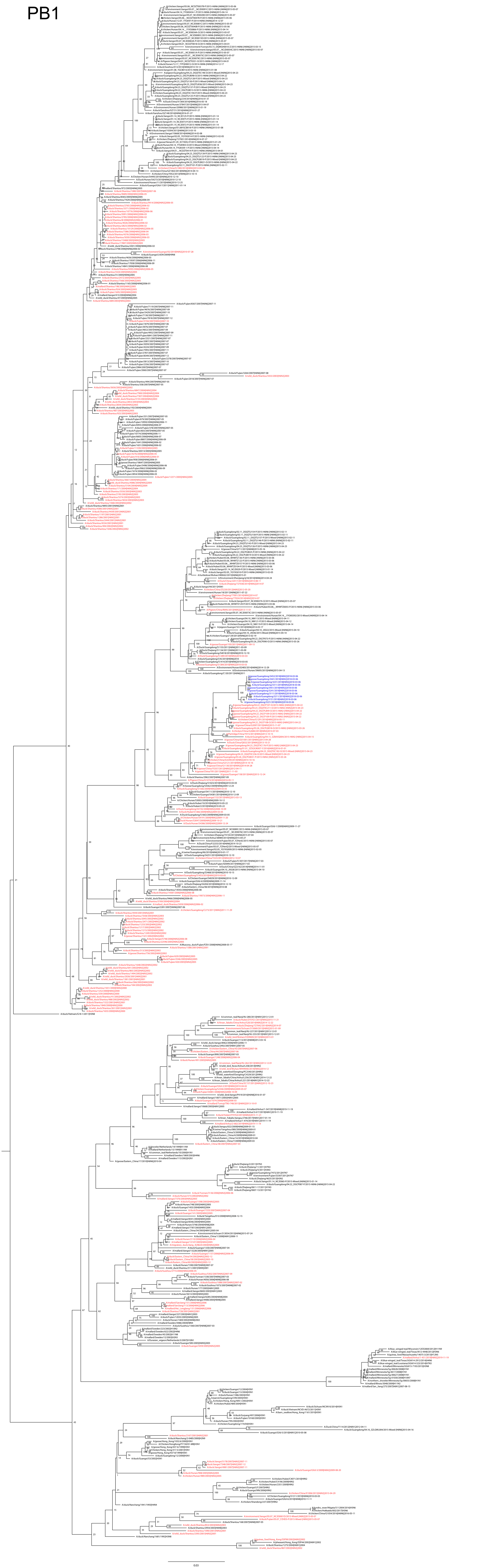

PA

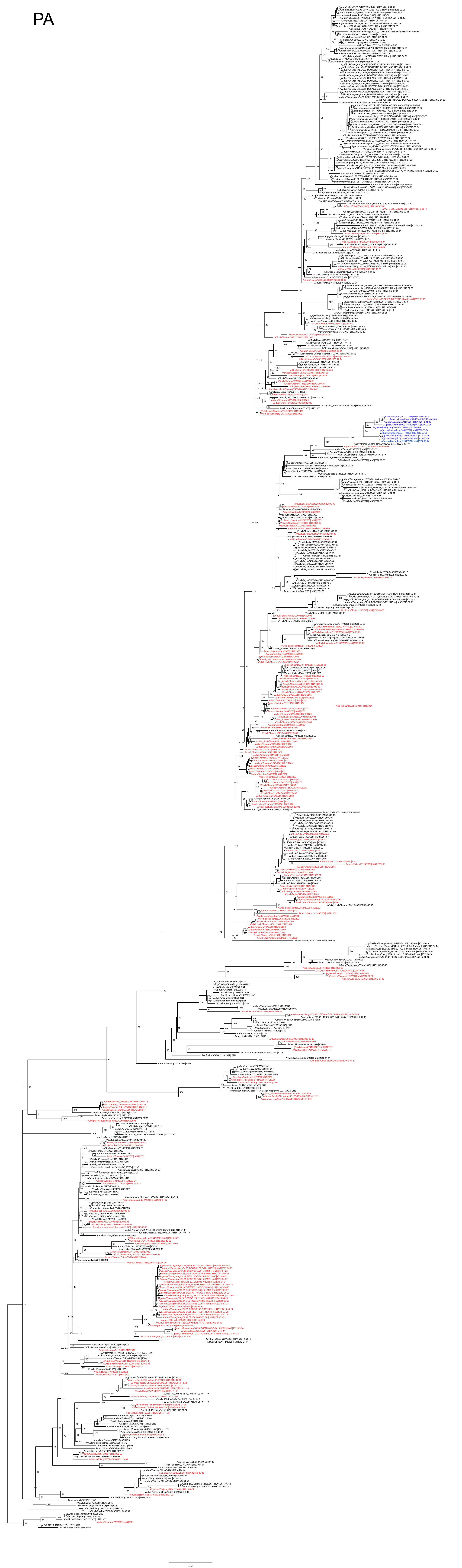

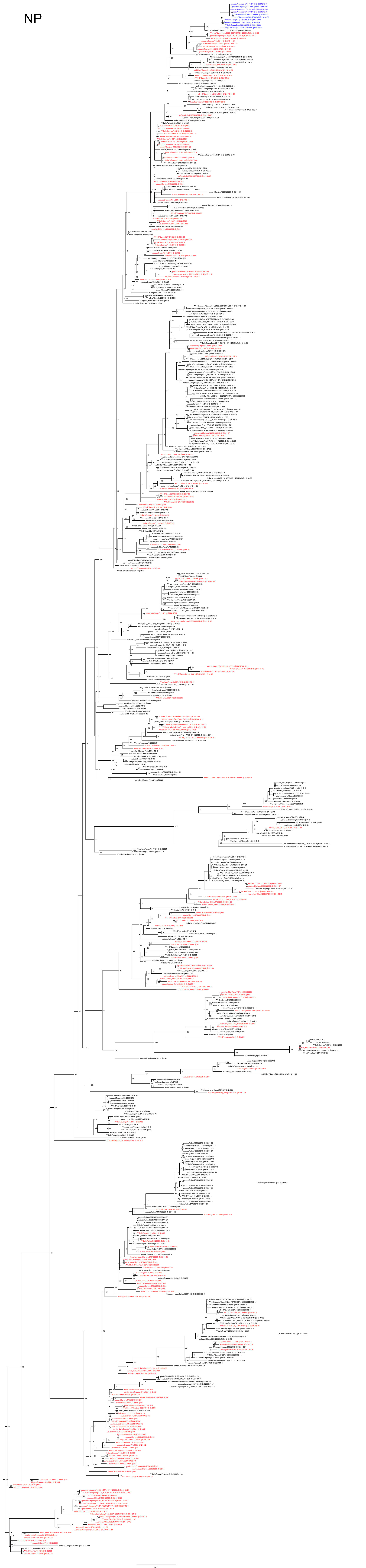
NP

M

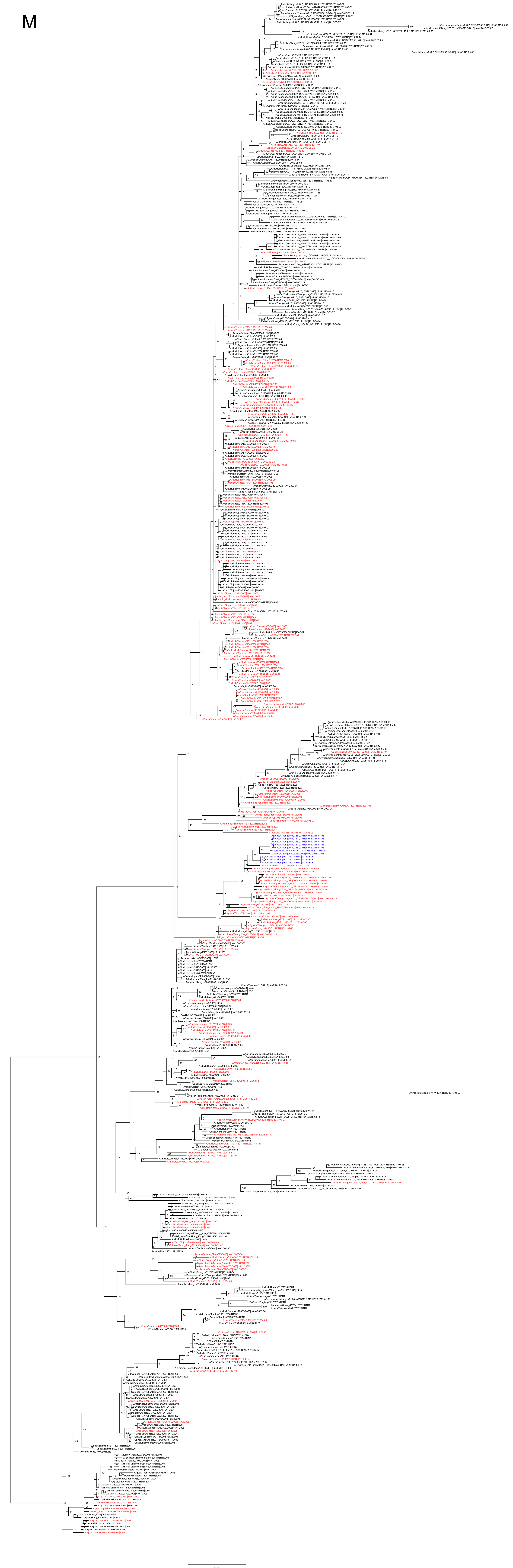

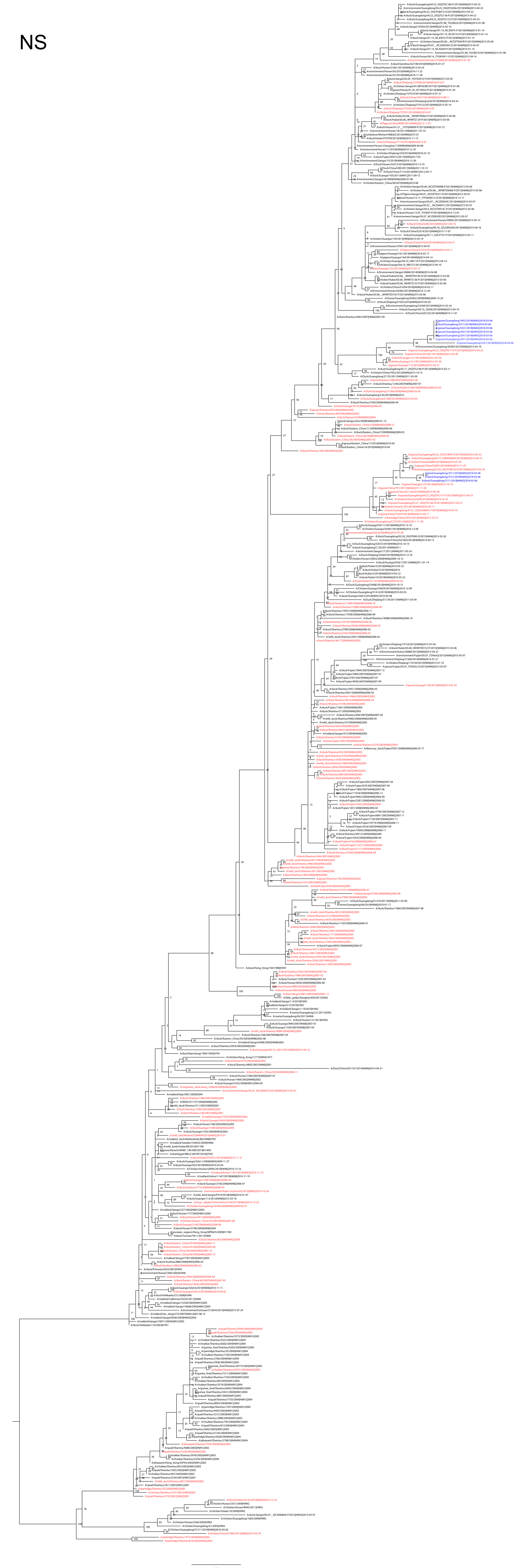
NS
0.02
