# Supplementary material for: The Genomic Evolution and the Transmission Dynamics of H6N2 Avian Influenza A Viruses in Southern China
Source: Viruses. 2022 May 26;14(6):1154. doi: 10.3390/v14061154 (PMC9229805; doi:10.3390/v14061154)
Supplement: Supplementary file 1 [file viruses-14-01154-s001.zip › Supplementary Figure S2.pdf]

Figure S2. MCC trees of the HA and the NA sequences of H6 subtype viruses sampled in China is shown. Shaded bars represent the 95% highest probability distribution for the age of each node

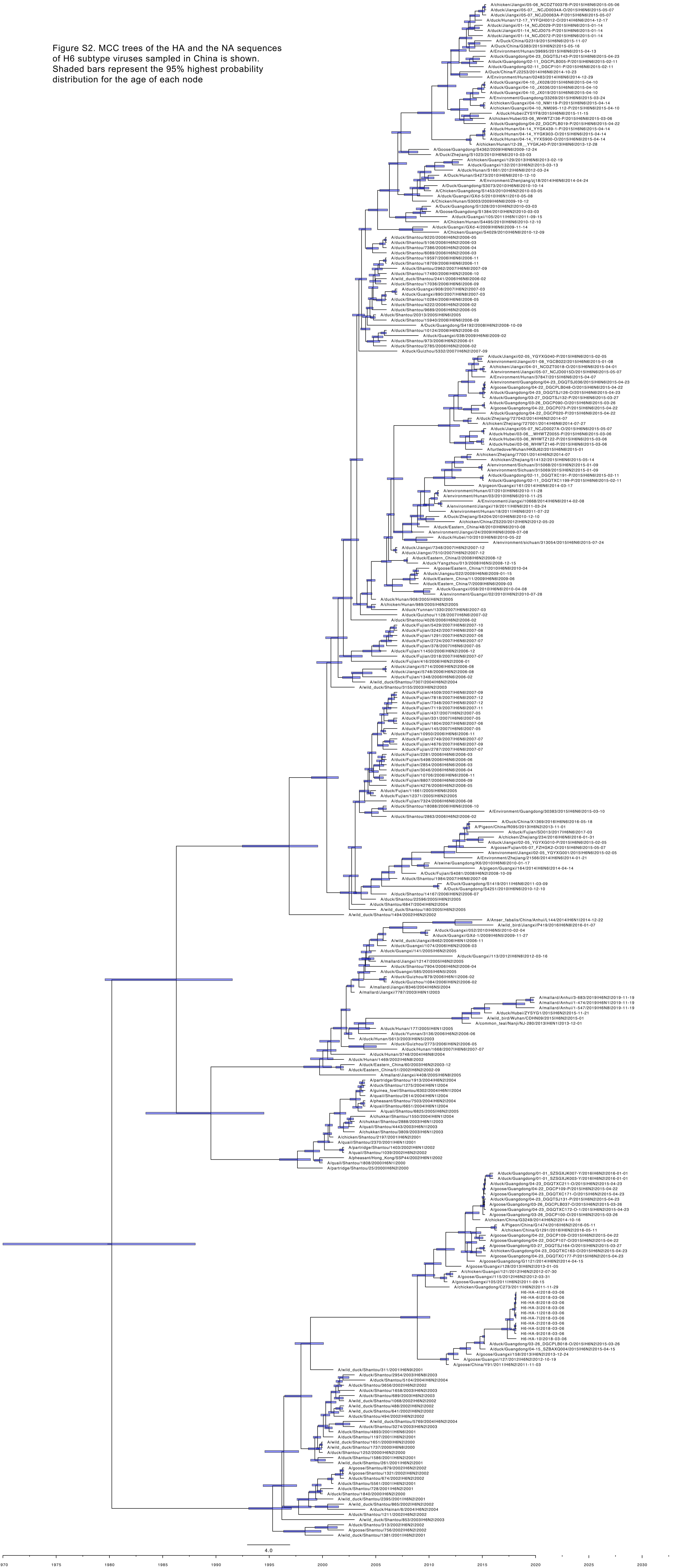

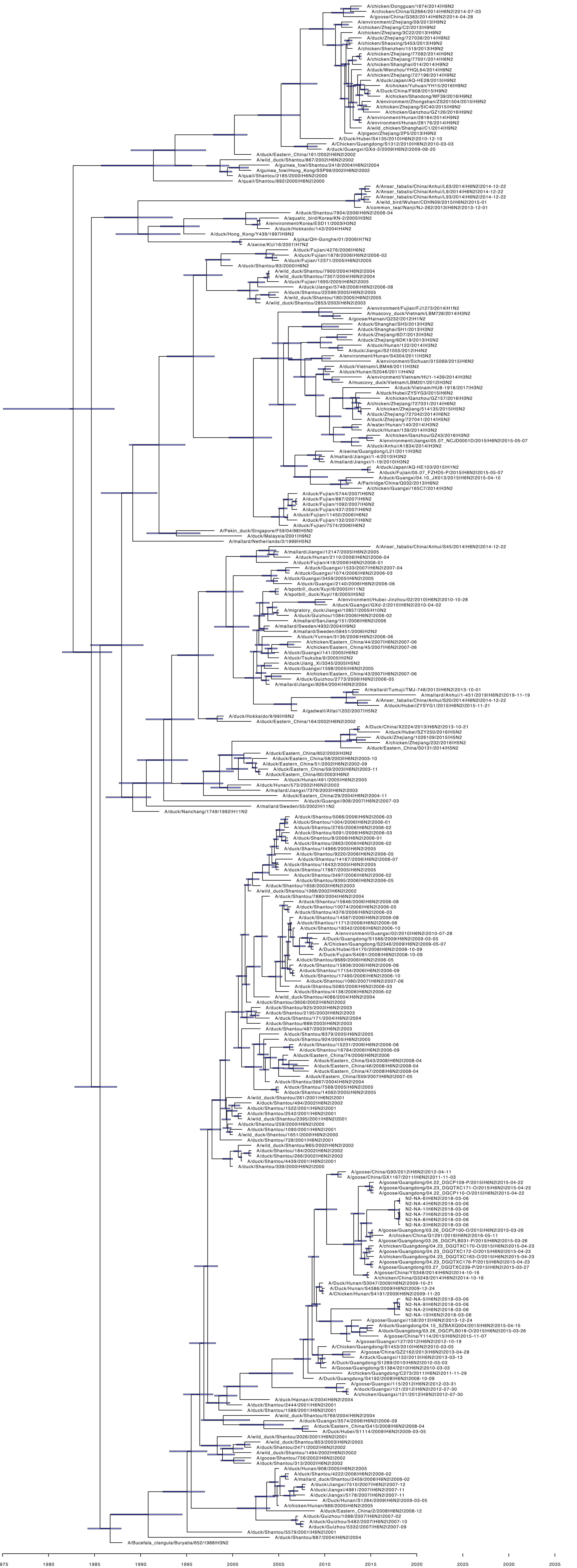
975
1980
1985
1990
1995
2000
2005
2010
2015
2020
2025
2030
2035
