# Supplementary material for: The Genomic Evolution and the Transmission Dynamics of H6N2 Avian Influenza A Viruses in Southern China
Source: Viruses. 2022 May 26;14(6):1154. doi: 10.3390/v14061154 (PMC9229805; doi:10.3390/v14061154)
Supplement: Supplementary file 1 [file viruses-14-01154-s001.zip › Supplementary Table S1.pdf]

Supplementary Table S1. The accession numbers of avian influenza A(H6N2) viruses in this study in GISAID database.

Strain name	Isolated ID	PB2	PB1	PA	HA	NP	NA	M	NS
A/duck/Guangdong /3231/2018(H6N2)	EPI_ISL_576280	EPI1805783	EPI1805784	EPI1805785	EPI1805786	EPI1805787	EPI1805788	EPI1805789	EPI1805790
A/duck/Guangdong /3111/2018(H6N2)	EPI_ISL_576374	EPI1805791	EPI1805792	EPI1805793	EPI1805794	EPI1805795	EPI1805796	EPI1805797	EPI1805798
A/goose/Guangdong /3451/2018(H6N2)	EPI_ISL_576375	EPI1805799	EPI1805800	EPI1805801	EPI1805802	EPI1805803	EPI1805804	EPI1805805	EPI1805806
A/goose/Guangdong /3441/2018(H6N2)	EPI_ISL_576376	EPI1805807	EPI1805807	EPI1805808	EPI1805809	EPI1805810	EPI1805811	EPI1805812	EPI1805813
A/duck/Guangdong /3311/2018(H6N2)	EPI_ISL_576378	EPI1805823	EPI1805824	EPI1805825	EPI1805826	EPI1805827	EPI1805828	EPI1805829	EPI1805830
A/goose/Guangdong /3241/2018(H6N2)	EPI_ISL_576379	EPI1805831	EPI1805832	EPI1805833	EPI1805834	EPI1805835	EPI1805836	EPI1805837	EPI1805838
A/goose/Guangdong /3452/2018(H6N2)	EPI_ISL_576380	EPI1805839	EPI1805840	EPI1805841	EPI1805842	EPI1805843	EPI1805844	EPI1805845	EPI1805846

A/duck/Guangdong /3151/2018(H6N2)	EPI_ISL_576381	EPI1805847	EPI1805848	EPI1805849	EPI1805850	EPI1805851	EPI1805852	EPI1805853	EPI1805854
A/goose/Guangdong /3221/2018(H6N2)	EPI_ISL_576382	EPI1805855	EPI1805856	EPI1805857	EPI1805858	EPI1805859	EPI1805860	EPI1805861	EPI18058362
