# Supplementary material for: The Genomic Evolution and the Transmission Dynamics of H6N2 Avian Influenza A Viruses in Southern China
Source: Viruses. 2022 May 26;14(6):1154. doi: 10.3390/v14061154 (PMC9229805; doi:10.3390/v14061154)
Supplement: Supplementary file 1 [file viruses-14-01154-s001.zip › Supplementary Table S2.pdf]

Supplementary Table 2. The lineage of H6N2 viruses in this study.

	PB2	PB1	PA	HA	NP	NA	M	NS
A/duck/Guangdong/3231/2018(H6N2)	Group I	Group III	Group I	Group I	Group III	Group I	Group I	Group I
A/duck/Guangdong/3111/2018(H6N2)	Group I	Group III	Group I	Group I	Group III	Group I	Group I	Group I
A/goose/Guangdong/3451/2018(H6N2)	Group I	Group III	Group I	Group I	Group III	Group I	Group I	Group I
A/goose/Guangdong/3441/2018(H6N2)	Group I	Group III	Group I	Group I	Group III	Group I	Group I	Group I
A/duck/Guangdong/3311/2018(H6N2)	Group I	Group III	Group I	Group I	Group III	Group I	Group I	Group I
A/goose/Guangdong/3241/2018(H6N2)	Group I	Group III	Group I	Group I	Group III	Group I	Group I	Group I
A/goose/Guangdong/3452/2018(H6N2)	Group I	Group III	Group I	Group I	Group III	Group I	Group I	Group I
A/duck/Guangdong/3151/2018(H6N2)	Group I	Group III	Group I	Group I	Group III	Group I	Group I	Group I
A/goose/Guangdong/3221/2018(H6N2)	Group I	Group III	Group I	Group I	Group III	Group I	Group I	Group I
